# Determining and predicting feed intake in crossbred sheep from weaning until maturity

**DOI:** 10.1007/s11250-023-03731-z

**Published:** 2023-09-23

**Authors:** P.G. Theron, T.S. Brand, S.W.P. Cloete, J.H.C van Zyl

**Affiliations:** 1 Department of Animal Sciences, Stellenbosch University, Private Bag X1, Matieland, 7602 South Africa; 2 Directorate: Animal Sciences, Department of Agriculture, Western Cape Government, Private Bag X1, Elsenburg, 7607 South Africa

**Keywords:** Feed conversion ratio, Feed efficiency, Feedlot, Growth, Intake models

## Abstract

Access to simple, accurate feed intake models would facilitate decision-making in feedlots as feed costs are a major part of operational expenditure. This study aimed to develop genotype-specific feed intake models for South African feedlot lambs. Four ram and four ewe lambs each of eight genotypes were raised under ideal growth conditions from weaning until 1 year of age. Feed intake and growth were monitored throughout this period. The intake data were then used to fit various models to predict daily feed intake, intake as percentage of body weight, cumulative intake and feed conversion ratio. No satisfactory univariate models could be found for the prediction of daily or percentage intake, but a good fit was found for cumulative intake data (*R*^2^ >0.80, *P* <0.01). The slope parameters of these linear models show a strong correlation (72%) with feed conversion and can therefore also serve as proxies for feed conversion. A model was also developed that can predict feed conversion ratio with a moderate accuracy (*R*^2^ =0.5, *P* <0.05) at a given body weight. The cumulative intake model was deemed accurate and simple enough for practical use.

## Report

As feed costs make up around 70% of the running cost in a feedlot (Lima et al. 2017; Zhang et al. 2017), feedlotters require accurate predictions of expected feed intake to aid in management and planning (Illius et al. 2000; Pulina et al. 2013). While there are models available for the prediction of voluntary feed intake (VFI) in sheep (Finlayson et al., 1995; Emmans 1997; National Research Council 2007; Pulina et al. 2013; Vieira et al. 2013), these models are generally complex and lack breed specificity. Previous studies have shown that significant differences exist between the feed intakes of different breeds (Hanset et al., 1987; Finlayson et al. 1995; van der Merwe et al. 2022), definitively confirming that a single, generalised model across breeds is unlikely to be fit for purpose. Genotype-specific models therefore need to be developed to enable producers to optimise their production enterprises as far as possible. This is a significant challenge in South Africa, where a wide array of breeds (SA Stud Book 2015; van der Merwe et al. 2020) can be found, and is further complicated by the use of crossbreeding in certain flocks.

This study therefore aimed to construct simple, practically applicable models for the prediction of feed intake between weaning and maturity in pure and crossbred South African sheep. It was expected that the best way of achieving this aim would be through the development of univariate models using readily available on-farm data such as live weight or age as predictive inputs as such models would be simple enough for broad uptake and use even by producers with limited scientific knowledge.

Ethical clearance for this study was granted by the Stellenbosch University REC:ACU (ACU-2020-14574). The project is briefly summarised below; for the full trial methodology, please see Theron (2021).

Four randomly selected groups of 20 Merino and 20 Dohne Merino ewes were mated to rams of their own breed or Dorper, Dormer or Ile de France rams to create two purebred control and six crossbred trial groups of offspring, namely purebred Dohne Merino, Dohne x Dorper, Dohne x Dormer, Dohne x Ile de France, purebred Merino, Merino x Dorper, Merino x Dormer and Merino x Ile de France. At weaning (100 days), four ram and four ewe lambs from each genotype were selected for the trial. They were housed in individual pens and adapted to a feedlot diet (16.38% CP, 11.44 MJ ME/kg) for a week. After adaptation, they had *ad libitum* access to the diet throughout the year-long trial period. Bodyweight and feed intake were recorded on a weekly basis throughout the trial, and 34 records per animal were collected (136 measurements per production group). Individual daily dry matter intakes (DFI), intake as a percentage of body weight and cumulative feed intake as well as feed conversion ratio (FCR) were calculated for the growth period.

Trial data was analysed using the non-linear estimation procedure in Statistica 14 (TIBCO Statistica 2020) where intake and feed conversion data were fitted to various equations (linear, quadratic, power) to find the best-fitting models. Both age and live weight were separately evaluated as inputs for the models. Table 1 provides the descriptive statistics and distribution of cumulative feed intake data for sex and genotypic groups, indicating that the data was normally distributed. The correlation between weight and feed intake for each group is also provided in the table. Since both weight and cumulative feed intake increase over time, a high positive correlation exists between these parameters.

**Table 1:** Descriptive statistics, data distribution and correlation coefficients between weight and cumulative feed intake for sex and genotype groups used in the trial

Group	*N*	Mean	Minimum	Maximum	SD	Skewness	Kurtosis	*r*
Ram	1060	205.836	3.298	490.240	126.573	0.135	−1.150	0.936
Ewe	1088	192.011	1.823	469.847	116.410	0.083	−1.181	0.943
Dohne Merino	265	193.178	5.790	414.104	114.277	0.049	−1.197	0.928
Dohne x Dorper	262	209.824	4.743	455.631	124.415	0.054	−1.189	0.978
Dohne x Dormer	272	221.169	4.686	469.847	133.624	0.043	−1.224	0.945
Dohne x Ile de France	271	204.870	7.360	490.240	127.647	0.166	−1.146	0.939
Merino	272	180.822	1.823	412.278	111.893	0.112	−1.205	0.916
Merino x Dorper	262	193.536	5.239	431.582	117.986	0.103	−1.147	0.957
Merino x Dormer	272	188.072	4.027	425.148	116.568	0.112	−1.190	0.897
Merino x Ile de France	272	199.282	4.197	468.736	122.275	0.123	−1.152	0.981

Throughout, it was found that body weight was a better predictor of feed intake than age. Daily dry matter intake followed a quadratic pattern relative to weight, concurring with results from previous studies (Lewis and Emmans 2020, 2010; van der Merwe et al. 2022). However, these models had low explicative value (*R*^2^ <0.249) and were deemed unsuitable for practical use. Greater success was achieved modelling intake as a percentage of body weight, with a linear relationship existing between the parameters. Between 32 and 57% of the variation in intake for the various genotypic groups was explained by the linear model, thus providing low to moderate predictive accuracy.

A linear model was found to best fit the cumulative intake data. Table 2 presents the model parameters for sex and genotype groups separately, since no interaction between sex and genotype was present. The *B* parameter differed between sexes, where ewes had a greater value than rams, indicating a higher rate of increase in feed consumption as the *B* parameter refers to the slope of a linear equation. This points to ewes having a poorer FCR than rams, as more feed is required per unit weight gained. Similar results were obtained by van der Merwe et al. (2022) for purebred sheep. This is probably due to ewes generally depositing fat at a greater rate than rams (van der Merwe et al. 2019) which leads to them having an increased maintenance requirement relative to rams (Brand et al. 2017).

**Table 2:** Parameter values as least squares means (±S.E.) of the linear fitting* of cumulative intake to weight for pure and crossbred lambs of both sexes from weaning until maturity

Main effects		Parameter		RMSE	*R*^2^
		*A*	*B*		
Sex	Ram	−268.201±10.517	6.631±0.157	44.384	0.876
	Ewe	−278.652±10.517	7.672±0.157	38.790	0.889
	*P*-value	*0.486*	*0.001*		
Genotype	Dohne Merino	−284.877±21.034	7.493±0.315	49.339	0.813
	Dohne x Dorper	−294.480±21.034	7.107±0.315	56.434	0.786
	Dohne x Dormer	−275.809±21.034	6.702±0.315	36.822	0.924
	Dohne x Ile de France	−257.354±21.034	6.867±0.315	42.535	0.889
	Merino	−290.365±21.034	7.803±0.315	43.593	0.848
	Merino x Dorper	−287.230±21.034	7.308±0.315	50.776	0.814
	Merino x Dormer	−239.534±21.034	6.958±0.315	33.010	0.920
	Merino x Ile de France	−257.764±21.034	6.974±0.315	33.610	0.924
	*P*-value	*0.533*	*0.258*		

*CI=A+B(*W*) where *CI* is cumulative intake, and *W* refers to body weight (both in kilogram)

With more than 82% of the variation in the intake data being explained, the models have a high predictive accuracy, provided that the input weights fall within the target range of 30–110 kg. The cumulative feed intake models are suitable for practical application as they have a potentially high degree of predictive accuracy and use body weight, which is regularly monitored in feedlots, as input factor. One hurdle to practical application is that the conversion of cumulative intake to DFI at any point may prove problematic. Since cumulative intake is plotted relative to weight gain, the rate of weight gain will influence DFI (van der Merwe et al. 2022). To calculate DFI based on the prediction of cumulative intake, the growth rate at that point will first have to be known. However, since growth models are available for the genotypes in this study (Theron 2021), this is achievable. The linear model also allows for a general comparison to be made between the FCR of various groups at different time points over a given time period. A correlation of 72% exists between the slope of the cumulative intake equation and the calculated average FCR over the growth period, thus allowing for a fairly accurate estimation of FCR.

Finally, the feed conversion ratios of the various groups were compared. The descriptive statistics and distribution of the feed conversion data are given in Table 3 for each production group as interactions between sex and genotype were present in this instance. This dataset was also normally distributed. Once again, the correlations between weight and feed intake are also given in the table. The correlation coefficients are lower than for cumulative feed intake due to FCR plateauing at a certain age and weight rather than continuing to increase.

**Table 3:** Descriptive statistics, data distribution and correlation coefficients between weight and FCR for production groups used in the trial

Genotype	Sex	*N*	Mean	Minimum	Maximum	SD	Skewness	Kurtosis	*r*
Dohne Merino	Ram	129	7.523	2.555	17.937	3.072	0.715	0.004	0.754
	Ewe	135	7.620	2.811	15.794	2.889	0.599	−0.500	0.690
Dohne x Dorper	Ram	125	6.945	1.416	14.904	3.009	0.627	−0.385	0.690
	Ewe	132	9.225	2.246	17.401	3.659	0.264	−1.029	0.765
Dohne x Dormer	Ram	136	7.352	1.675	17.001	2.955	0.605	0.000	0.765
	Ewe	136	7.365	2.281	13.326	2.319	−0.015	−0.501	0.721
Dohne x Ile de France	Ram	135	7.207	1.872	13.666	2.894	0.229	−0.911	0.721
	Ewe	136	7.832	2.855	15.369	3.253	0.525	−0.903	0.695
Merino	Ram	135	7.867	1.538	16.304	3.684	0.452	−0.737	0.695
	Ewe	136	7.917	1.008	16.696	3.294	0.618	0.078	0.801
Merino x Dorper	Ram	126	7.384	2.351	16.793	3.186	0.592	−0.454	0.801
	Ewe	129	9.017	2.794	17.427	3.739	0.437	−0.741	0.780
Merino x Dormer	Ram	136	6.834	2.502	14.101	2.534	0.508	−0.460	0.780
	Ewe	134	8.877	2.090	17.912	4.079	0.536	−0.847	0.754
Merino x Ile de France	Ram	136	6.870	1.753	13.000	2.359	0.225	−0.272	0.754
	Ewe	134	8.082	2.854	17.274	3.086	0.807	0.389	0.684

A power equation (*FCR* = *A* × *Weight*^*B*^) provided the best fit for the data, and results from this modelling are provided in Table 4. Approximately 55% of the variation in FCR could be explained by the fitting of the model.

**Table 4:** Parameter values as least squares means (±S.E.) of the power equation (*FCR* = *A* × *Weight*^*B*^) fitted to body weight to predict FCR for pure and crossbred lambs of both sexes from weaning until maturity

Genotype	Sex	Parameter		RMSE	*R*^2^
		*A*	*B*		
Dohne Merino	Ram	0.0263^e^ ±0.0337	1.384^bc^ ±0.109	2.001	0.573
	Ewe	0.0641^bcde^±0.0337	1.193^bcdef^ ±0.109	2.111	0.488
Dohne x Dorper	Ram	0.0421^de^ ±0.0337	1.300^bcde^ ±0.109	2.111	0.504
	Ewe	0.0293^de^ ±0.0337	1.451^ab^ ±0.109	1.882	0.584
Dohne x Dormer	Ram	0.0327^de^ ±0.0337	1.267^bcde^ ±0.109	1.882	0.592
	Ewe	0.2600^a^ ±0.0337	0.806^g^ ±0.109	1.994	0.541
Dohne x Ile de France	Ram	0.1224^bcd^ ±0.0337	1.071^defg^ ±0.109	1.994	0.522
	Ewe	0.0292^de^ ±0.0337	1.355^abcd^ ±0.109	2.641	0.638
Merino	Ram	0.0477^cde^ ±0.0337	1.386^abc^ ±0.109	2.641	0.482
	Ewe	0.1400^cb^ ±0.0337	1.122^cdef^ ±0.109	1.877	0.478
Merino x Dorper	Ram	0.0332^de^ ±0.0337	1.374^abcd^ ±0.109	1.877	0.651
	Ewe	0.0310^de^ ±0.0337	1.440^ab^ ±0.109	1.580	0.523
Merino x Dormer	Ram	0.0982^bcde^±0.0337	1.038^efg^ ±0.109	1.580	0.608
	Ewe	0.0146^e^ ±0.0337	1.628^a^ ±0.109	1.545	0.687
Merino x Ile de France	Ram	0.1567^b^ ±0.0337	0.898^fg^ ±0.109	1.545	0.568
	Ewe	0.0496^cde^ ±0.0337	1.291^bcde^ ±0.109	2.061	0.475
*P*-value	Genotype	*0.018*	*0.014*		
	Sex	*0.663*	*0.198*		
	Interaction	*<0.001*	*<0.001*		

Means with different superscripts in the same column (a-e) differ significantly (*P* ≤ 0.05)

The purebred Merino group displayed the least favourable FCR, indicating that it is the least suitable for feedlot finishing of the genotypes included in the study. Most of the other groups displayed fairly similar FCR values until 60–70 kg, whereafter more prominent variations began occurring. Feed conversion ratio differed between rams and ewes as indicated by the slope parameter of the cumulative intake equation and previous studies (Kashan et al. 2005; Kashani and Bahari 2017; van der Merwe et al. 2022). Crossbreeding did not provide a universal improvement in FCR. The crossbred Merino genotypes displayed an improvement in FCR relative to purebred Merinos, but only the Dohne x Dormer group displayed a better FCR than the purebred Dohne Merinos. This contrast is supported by literature as no universal trend is reported. Kashan et al. (2005) and Schiller et al. (2015) reported that feed conversion improved in crossbred lambs, while Kiyanzad (2002) and Khaldari and Ghiasi (2018) did not find any improvement.

In conclusion, it can be stated that the objective of creating practically applicable models to predict feed intake for the genotypes included in this study has been achieved with the development of linear cumulative intake models. These cumulative intake models also allowed for an estimation of FCR over the entire growth period and the relative ranking of groups for feed efficiency. It appears that no significant differences exist between these genotypic groups with regard to feed intake and that crossbreeding did not provide universal improvements in FCR.

## Data Availability

The data sets generated during the current study are available from the corresponding author upon reasonable request.
